# Investigation of Polyamine Metabolism and Homeostasis in Pancreatic Cancers

**DOI:** 10.3390/medsci5040032

**Published:** 2017-12-07

**Authors:** Chelsea Massaro, Jenna Thomas, Otto Phanstiel

**Affiliations:** Department of Medical Education, College of Medicine, University of Central Florida, Orlando, FL 32826-3227, USA; clm12d@my.fsu.edu (C.M.); jenna.thomas97@Knights.ucf.edu (J.T.)

**Keywords:** polyamine, cancer, metabolism, difluoromethylornithine, polyamine transport inhibitor, pancreatic ductal adenocarcinoma

## Abstract

Pancreatic cancers are currently the fourth leading cause of cancer-related death and new therapies are desperately needed. The most common pancreatic cancer is pancreatic ductal adenocarcinoma (PDAC). This report describes the development of therapies, which effectively deplete PDAC cells of their required polyamine growth factors. Of all human tissues, the pancreas has the highest level of the native polyamine spermidine. To sustain their high growth rates, PDACs have altered polyamine metabolism, which is reflected in their high intracellular polyamine levels and their upregulated import of exogenous polyamines. To understand how these cancers respond to interventions that target their specific polyamine pools, L3.6pl human pancreatic cancer cells were challenged with specific inhibitors of polyamine biosynthesis. We found that pancreatic cell lines have excess polyamine pools, which they rebalance to address deficiencies induced by inhibitors of specific steps in polyamine biosynthesis (e.g., ornithine decarboxylase (ODC), spermidine synthase (SRM), and spermine synthase (SMS)). We also discovered that combination therapies targeting ODC, SMS, and polyamine import were the most effective in reducing intracellular polyamine pools and reducing PDAC cell growth. A combination therapy containing difluoromethylornithine (DFMO, an ODC inhibitor) and a polyamine transport inhibitor (PTI) were shown to significantly deplete intracellular polyamine pools. The additional presence of an SMS inhibitor as low as 100 nM was sufficient to further potentiate the DFMO + PTI treatment.

## 1. Introduction

Pancreatic ductal adenocarcinoma (PDAC) typically develops slowly, over many years, from precursor pancreatic intraepithelial neoplasias (PanINs) [1,2]. With a five-year survival rate of less than 8%, new therapies are desperately needed to address this fourth leading cause of cancer-related death. The most frequently mutated gene in human PDAC is KRAS (95%) and human PDAC cell lines often (50%) have a copy number gain in MYC (c-myc) [3]. Since the MYC gene family is activated in nearly 70% of all human cancers, targeting myc-driven processes could have broad applications. Both KRAS and MYC are upstream activators of polyamine metabolism, and their mutations are known to increase intracellular polyamine levels to presumably drive tumor growth [4].

**Figure 1:**
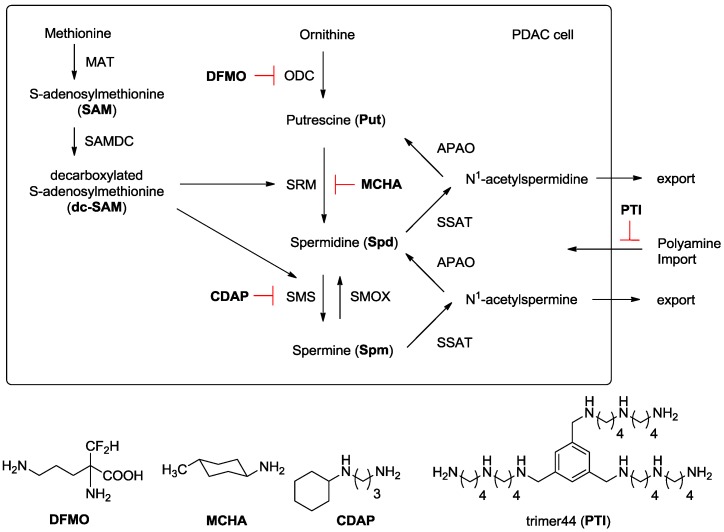
Polyamine Metabolism and Transport. Ornithine is converted to putrescine via ornithine decarboxylase (ODC). Methionine is converted to S-adenosylmethionine (SAM) via methionine adenosyltransferase (MAT) and SAM is converted to decarboxylated *S*-adenosylmethionine (dc-SAM) via the action of *S*-adenosylmethionine decarboxylase (SAMDC). Putrescine is converted to spermidine via spermidine synthase (SRM) and an aminopropyl fragment derived from dc-SAM. Similarly, spermidine is converted to spermine via spermine synthase (SMS) and dc-SAM. Back conversion can occur via *N*-acetylation using spermidine/spermine *N*^1^-acetyltransferase (SSAT) to form *N*^1^-acetyl derivatives, which can be oxidized by acetylpolyamine oxidase (APAO) to generate the respective polyamine. *N*^1^-acetylpolyamines can also be excreted by cells to maintain intracellular polyamine levels and exogenous polyamines can be imported to increase intracellular polyamine pools via the polyamine transport system. Spermine oxidase (SMOX) allows direct conversion of spermine to spermidine. PDAC: pancreatic ductal adenocarcinoma; DFMO: difluoromethylornithine; MCHA: *trans*-4-methyl-cyclohexylamine; CDAP: *N*-cyclohexyl-1,3-diaminopropane; PTI: polyamine transport inhibitor.

At physiological pH, the native polyamines exist as polycations and can interact with anionic biomolecules like RNA, DNA, and proteins. As a result, polyamines have pleiotropic effects in cells and play critical roles in chromatin remodeling, transcription, translation, eIF5A activation, potassium channel regulation, and in the immune response [4]. The normal pancreas has the highest level of the native polyamine spermidine (Spd) of any mammalian tissue [5,6,7]. Our preliminary investigations revealed that PDAC cells are addicted to polyamines and have high intracellular polyamine levels and upregulated polyamine import activity [8,9]. Polyamines are charged and exist in millimolar levels inside cells, and maintaining polyamine homeostasis is critical to cell viability. As shown in Figure 1, homeostasis is tightly regulated via a balance between polyamine biosynthesis, catabolism, and transport.

This report investigated how PDAC cells respond to inhibition of discrete steps in polyamine biosynthesis and importation. While homeostatic control was expected where polyamine pools could be interconverted to replenish a specific polyamine deficiency, it was unclear as to which specific enzyme (or combination of enzymes) needed to be inhibited to affect PDAC cell growth. Understanding this paradigm is important as it provides an opportunity to design new therapies to treat these deadly cancers by effectively targeting their enhanced reliance upon polyamine growth factors. Specific inhibitors for the polyamine biosynthetic enzymes ornithine decarboxylase (ODC), spermidine synthase (SRM), and spermine synthase (SMS) are known: these are α-difluoromethylornithine (DFMO) [2], trans-4-methylcyclohexylamine (MCHA) [10], and *N*-cyclohexyl-1,3-diaminopropane (CDAP) [11], respectively (Figure 1). Our group also developed a polyamine transport inhibitor (PTI, trimer44, Figure 1), which competitively inhibits import of the native polyamines and works synergistically with DFMO to inhibit cell growth in vitro [9,12]. In this manner, we were able to block both polyamine biosynthesis and importation, and decrease specific intracellular polyamine pools.

By using single agents and combinations of these compounds, we investigated how these interventions affected pancreatic cancer cell growth and intracellular polyamine pools.

## 2. Materials and Methods

### 2.1. Materials

DFMO was obtained as a gift from Dr. Patrick Woster at the Medical University of South Carolina. The inhibitors MCHA and CDAP were obtained from Acros Organics (Fair Lawn, NJ, USA) and Alfa Aesar (Ward Hill, MA, USA), respectively. The trimer44NMe PTI was prepared as previously described [9]. The compounds were readily soluble in water and stock solutions were made in phosphate buffered saline (PBS).

### 2.2. Biological Studies

L3.6pl cells were grown in Roswell Park Memorial Institute (RPMI) 1640 medium supplemented with 10% fetal bovine serum (FBS) and 1% penicillin/streptomycin. The cells were grown at 37 °C under a humidified 5% CO_2_ atmosphere. Cells in early- to mid-log phase were used.

### 2.3. IC_50_ Determinations and Cell Growth Studies

Cell growth assays were performed in sterile 96-well microtiter plates (Costar 3599, Corning, NY, USA). L3.6pl cells were seeded at 500 cells/70 µL with 250 µM aminoguanidine in each well. Aminoguanidine was added to prevent the oxidation of polyamine compounds by the bovine serum amine oxidase present in the FBS. Drug solutions (10 µL per well) of appropriate concentration in PBS were added after overnight incubation of each cell line. When necessary, PBS was added to make the total volume 100 µL in each well. After drug exposure for 72 h at 37 °C, cell growth was determined by measuring formazan formation from the 3-(4,5-dimethylthiazol-2-yl)- 5-(3-carboxymethoxyphenyl)-2-(4-sulfenyl)-2*H*-tetrazolium inner salt (MTS) using a SynergyMx Biotek microplate reader measuring absorbance at 490 nm [13]. All experiments were run in triplicate and compared to untreated controls.

### 2.4. HPLC and Polyamine Level Determination

L3.6pl cells (500,000 cells/10 mL media) were incubated with aminoguanidine (250 µM) at 37 °C for 24 h. Each compound (dissolved in PBS) was then added either alone or in combination with other agents, and the cells were incubated for 72 h at 37 °C. The cells were then washed extensively with ice cold PBS (once with 5 mL and twice with 2 mL). Each PBS wash was removed by suction. To the washed cells, an additional 2 mL of ice cold PBS was added and the cells were scraped off the dish and collected in a centrifuge tube. This scraping step was done twice for each experimental condition. The cell suspensions were then centrifuged at 1000 rpm for 4 min to provide a cell pellet. The supernatant was carefully removed by suction. The cell pellet was then lysed using a 0.2 M perchloric acid/1 M NaCl solution (200 µL), sonicated, and centrifuged. The resultant supernatant and pellet were separated. To 100 µL of the polyamine-containing supernatant 1,7-diaminoheptane (30 µL of 1.5 × 10^−4^ M) was added as an internal standard. The supernatant was then treated on the rotary shaker at 200 rpm for 1 h at 65 °C with 1 M Na_2_CO_3_ (200 µL) and dansyl chloride (5 mg/mL, 400 µL) to generate the respective *N*-dansylated polyamines. Proline (1 M, 100 µL) was added and the reaction mixture shaken on the rotary shaker at 200 rpm for 20 min at 65 °C. The dansylated polyamines were then extracted into chloroform (1 mL) and the organic layer separated and concentrated. The resulting residue was redissolved in MeOH (1 mL) and the solution filtered through hydrophobic reversed phase C_18_ cartridges (50 mg bed weight). Each polyamine was then quantified via HPLC analysis [14] using authentic standards. The protein content of the pellet was quantified using the Pierce BCA Protein Assay Kit from ThermoFisher Scientific (Waltham, MA, USA). Final results were expressed as nmol polyamine/mg protein. Each condition was performed in duplicate.

### 2.5. Statistical Analysis

All cell growth studies were performed in triplicate. Polyamine level determination studies were performed in duplicate. Analyses were done using an unpaired *t*-test on GraphPad Prism 7 (GraphPad Software, San Diego, CA, USA). In all cases, values of *p* < 0.05 were regarded as being statistically significant.

## 3. Results

### Bioevaluation

**Figure 2:**
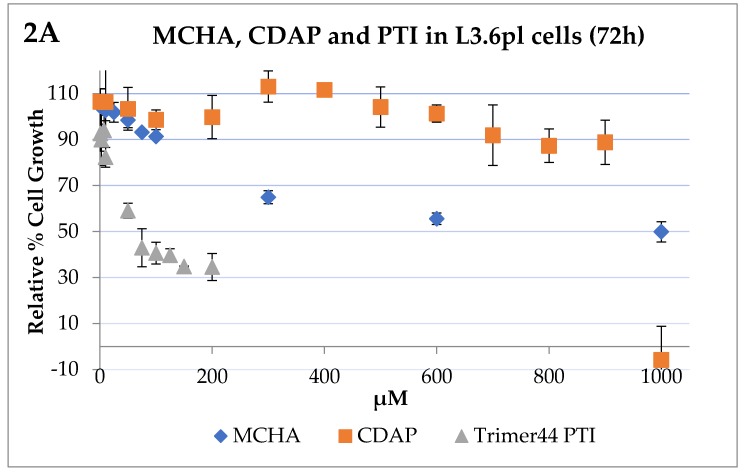

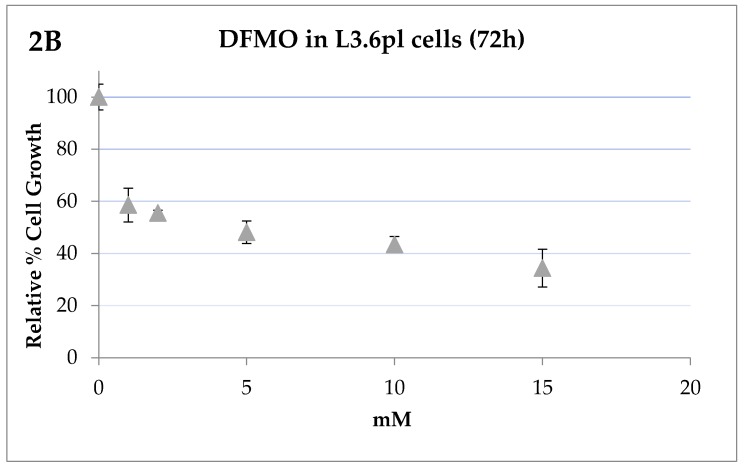
Influence of trans-4-methylcyclohexylamine (MCHA), *N*-cyclohexyl-1,3-propanediamine (CDAP), polyamine transport inhibitor (PTI, trimer 44), and difluoromethylornithine (DFMO) on L3.6pl human pancreatic cancer cell growth. Panel A: Growth inhibition by MCHA, CDAP, and the trimer44 PTI in L3.6pl cells after 72 h incubation at 37 °C in a 5% CO_2_ atmosphere. The 72 h IC_50_ values of MCHA, CDAP, and trimer44 PTI in L3.6pl human pancreatic cancer cells were 1000 ± 4.4 µM, 941 ± 12.1 µM, and 69.6 ± 1.8 µM, respectively. In general, the PTI was well tolerated at low doses (e.g., 4 µM with no reduction in growth) and the other inhibitors could be dosed at high concentrations with little effect on cell growth. For example, after 72 h, MCHA and CDAP at 100 µM gave 91% and 99% relative growth, respectively, versus the untreated control. Panel B: Relative cell growth in the presence of DFMO in L3.6pl cells after 72 h incubation at 37 °C in a 5% CO_2_ atmosphere. The 72 h IC_50_ value of DFMO in L3.6pl cells was 4.24 ± 0.11 mM.

A previous investigation of a series of pancreatic cancer cell lines identified the human L3.6pl cell line as an excellent model to look at polyamine metabolism and import due to its high polyamine transport activity [8]. A dose–response curve was obtained for each compound tested as a single agent to determine the IC_50_ value: the dose at which the growth of cells was inhibited 50% compared to the untreated control. We observed that L3.6pl cells (500 cells/well with 250 µM aminoguanidine) became more sensitive to DFMO over time (i.e., after 48 h, 72 h, and 96 h of incubation at 37 °C). The 72 h incubation time was selected to balance the cells’ DFMO sensitivity and the ability of these DFMO-treated cells to be rescued back with exogenous spermidine (1 µM) to ≥90% of the growth observed with the untreated control. The IC_50_ value of DFMO was 4.2 mM after 72 h incubation and these cells could be rescued back to ≥90% of the growth observed with the untreated control by Spd (1 µM) [8]. We also screened MCHA, CDAP and the trimer44 PTI for their ability to affect L3.6pl cell growth as single agents after 72 h of incubation (Figure 2). As shown in Figure 2, both MCHA and CDAP were relatively non-toxic and required high concentrations to affect L3.6pl cell growth. The L3.6pl 72 h IC_50_ value of PTI trimer44 was 69.6 ± 1.8 µM and typically the trimer44 alone could be dosed at 4 µM with no effect on cell growth.

**Figure 3:**
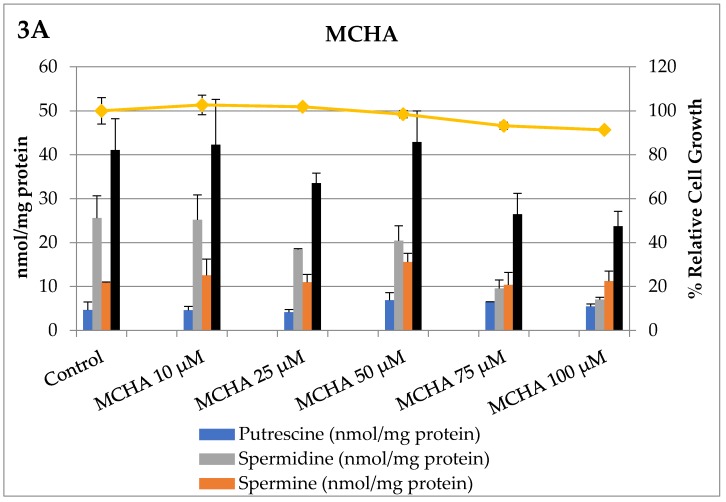

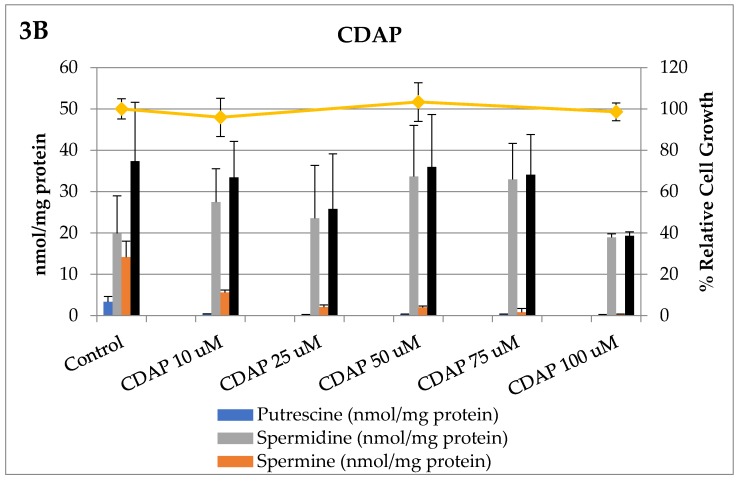
The effects of MCHA (Panel A) and CDAP (Panel B) on intracellular polyamine pools of L3.6pl cells after 72 h incubation. As shown in the top panel, the SRM inhibitor, MCHA, reduced intracellular spermidine pools in a dose-dependent manner and at 100 µM resulted in a 73% reduction of intracellular spermidine pools (gray bars, *p* < 0.05) and provided an overall 42% reduction of total intracellular polyamine pools (black bars). In the bottom panel, the SMS inhibitor, CDAP, reduced intracellular spermine pools in a dose-dependent manner and at 100 µM resulted in a 99.9% reduction in intracellular spermine levels and provided a 48% reduction of total intracellular polyamine pools. The reduction in spermine levels was statistically significant (*p* < 0.05) for concentrations of CDAP greater than 10 µM compared to untreated controls. Neither intervention significantly reduced relative cell growth (vs. untreated controls), consistent with these cells having excess polyamine pools.

Armed with this knowledge of how L3.6pl cells responded to these compounds as single agents, we explored how MCHA and CDAP affected intracellular polyamine pools in a concentration-dependent manner. These results are shown in Figure 3.

**Figure 4:**
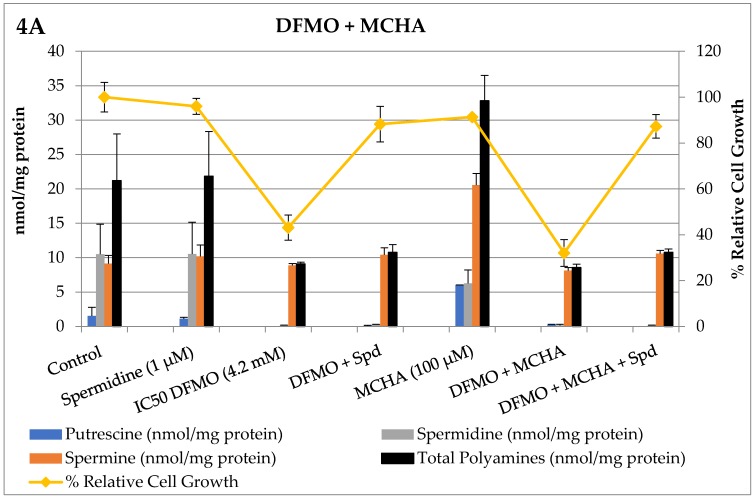

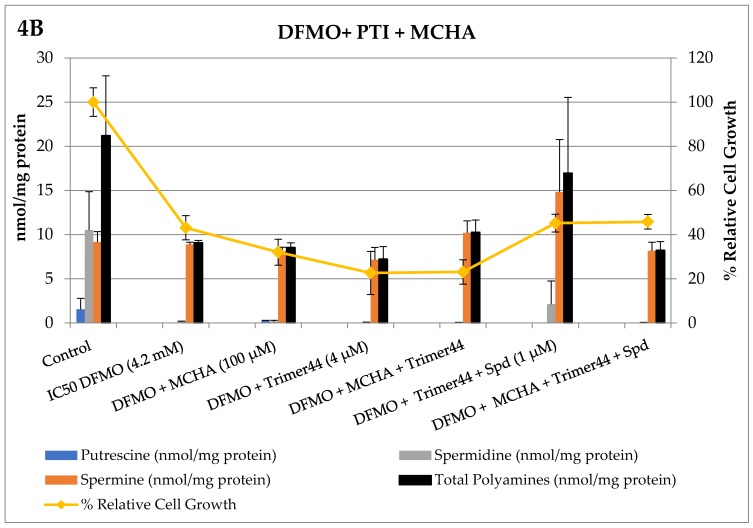
Single and combination therapies in L3.6pl cells with the spermidine synthase inhibitor, MCHA, at 100 µM. Altered polyamine pools (expressed as nmoles polyamine/mg protein) and L3.6pl relative % cell growth (vs. an untreated control) were observed after 72 h incubation. Controls were run in parallel, polyamine levels were determined in duplicate, and % cell growth determined in triplicate. The concentrations of compounds were: DFMO (4.2 mM), MCHA (100 µM), trimer44 PTI (4 µM), and spermidine (Spd, 1 µM). At these doses only DFMO gave significant reductions in cell growth when tested alone. In Panel A, the reduction in putrescine and spermine was statistically significant (*p* < 0.05) compared to untreated controls. Relative cell growth tracked fairly well with total intracellular polyamine pools.

**Figure 5:**
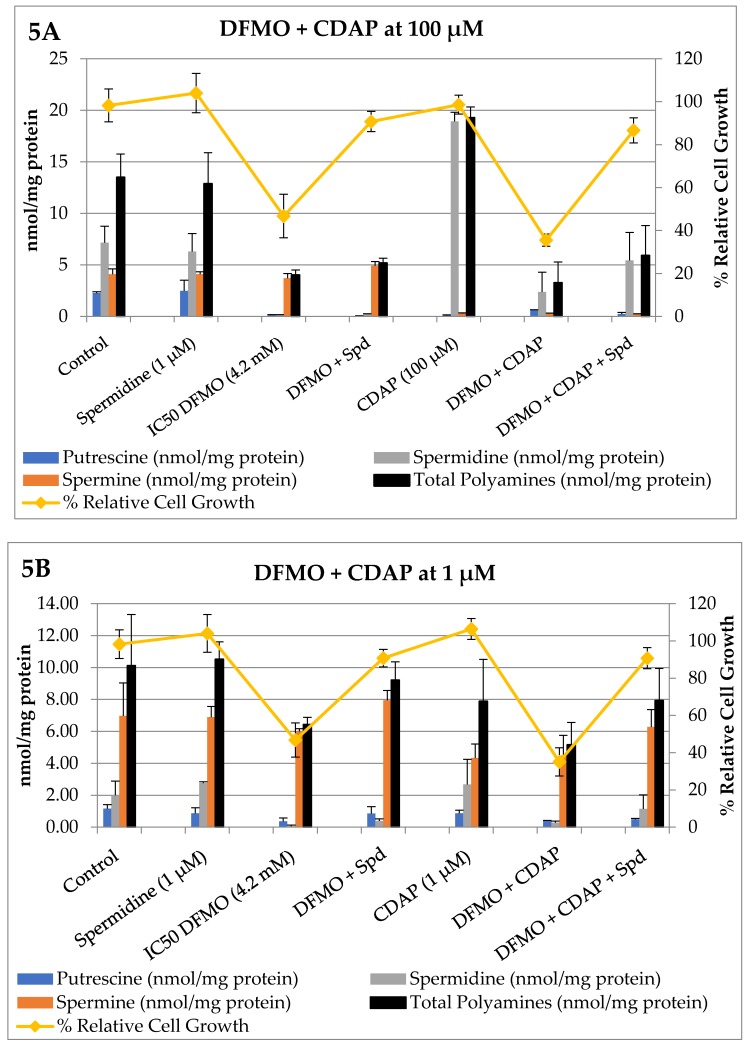

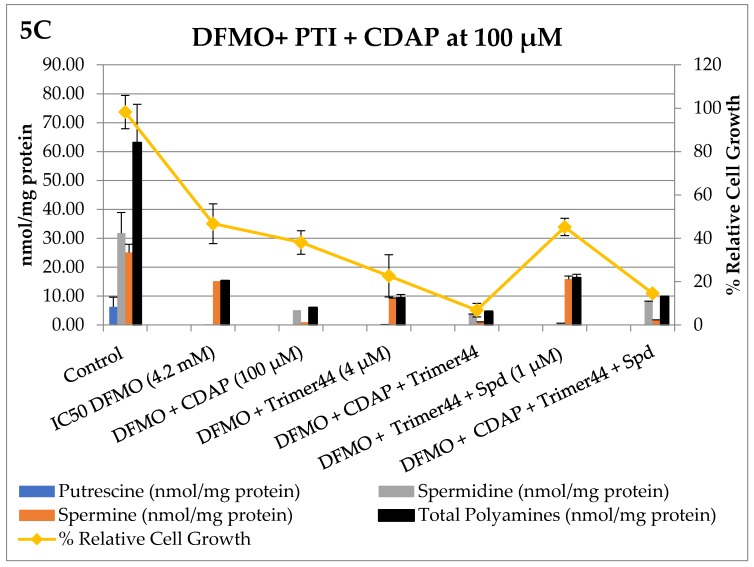
Single and combination therapies in L3.6pl cells with the spermine synthase inhibitor, CDAP. Altered polyamine pools (expressed as nmoles polyamine/mg protein) and L3.6pl relative % cell growth (vs. an untreated control) were observed after 72 h incubation. Controls were run in parallel, polyamine levels were determined in duplicate, and % cell growth in triplicate. The concentrations of compounds were: DFMO (4.2 mM), CDAP (100 µM in Panels A and C and 1 µM in Panel B), trimer44 PTI (4 µM), and spermidine (Spd, 1 µM). Note: in Panel A, we also tested CDAP (100 µM) + Spd (1 µM), which gave 99% relative cell growth and 0.08 ± 0.20, 13.72 ± 0.85, 0.20 ± 0.09, and 14.00 ±0.97 nmol/mg protein for putrescine, spermidine, spermine, and total polyamines, respectively (not shown). This revealed that exogenous spermidine (1 µM) did not further enhance intracellular spermidine levels in the presence of CDAP (100 µM). This observation is consistent with homeostatic control of polyamine levels. In contrast, in the presence of DFMO (4.2 mM), CDAP (100 µM), and exogenous spermidine (1 µM), significant rescue as evidenced by increased cell growth (from 35% to 86%) was observed, which is consistent with the observed increase in intracellular spermidine pools.

Next, we measured how DFMO and the PTI (trimer44) modulated polyamine pools (Table 1 and Table 2), and then tested how CDAP and MCHA, when tested individually in combination with DFMO or DFMO + PTI, affected intracellular polyamine levels and % relative cell growth. These 72 h experiments were conducted in L3.6pl cells and the results are shown in Figure 4 and Figure 5.

As shown in Figure 4 Panel A, only DFMO alone (43% cell growth, 4.2 mM) and in combination with MCHA (32%, 100 µM) resulted in significantly reduced cell growth. Exogenous spermidine (1 µM) was shown to rescue the growth of DFMO and DFMO + MCHA treated L3.6pl cells. As shown in Figure 4 Panel B, the presence of either MCHA, the trimer44 PTI, or the combination of MCHA + PTI with DFMO decreased cell growth. The combination of DFMO + MCHA + PTI + Spd resembled the DFMO + MCHA control, which is consistent with the trimer44 PTI inhibiting spermidine import. Indeed, the presence of the PTI prevented spermidine rescue because L3.6pl cells treated with DFMO + MCHA + Spd gave 87% relative cell growth, whereas cells treated with DFMO + MCHA + PTI + Spd gave 46% relative cell growth and gave nearly identical intracellular polyamine pools to the DFMO + MCHA entry.

Having surveyed MCHA and its effectiveness in combination with DFMO and the trimer44 PTI, we next evaluated CDAP and the results are shown in Figure 5.

As shown in Figure 5 Panel A, exogenous spermidine (1 µM) alone had little effect on intracellular polyamine pools and the growth of L3.6pl cells, presumably due to homeostatic controls which maintain preferred polyamine levels. In contrast, as seen in Panel A, DFMO (4.2 mM) resulted in 47% relative growth and a significant loss of intracellular putrescine and spermidine pools. DFMO-treated cells were rescued with exogenous spermidine (1 µM), which resulted in 90% relative growth and increased spermine pools. Interestingly, as will be illustrated again later in the discussion, these cells had a potential choice between living on spermidine or spermine, and elected to maintain spermine levels by shunting the imported spermidine to spermine. This commitment to spermine maintenance is consistent with previous findings [4,11].

CDAP at 100 µM was very effective in depleting ~90% and 95% of intracellular putrescine and spermine pools in L3.6pl cells after 72 h. As shown in Figure 5 Panel A, CDAP (100 µM)-treated L3.6pl cells increased their total intracellular polyamine pools via a 2.6-fold increase in intracellular spermidine levels. Interestingly, these high spermidine levels could not be driven higher in the presence of exogenous spermidine (1 µM), suggesting a possible upper limit under these conditions (see legend of Figure 5). The combination of CDAP (100 µM) and DFMO (4.2 mM) resulted in decreased cell growth and decreased intracellular spermidine pools. Both cell growth and spermidine pools could be rescued from DFMO + CDAP treatment by the addition of exogenous spermidine (1 µM). These results illustrate the dynamic exchange between polyamine pools and the plasticity of the homeostatic system to inhibitors of polyamine biosynthesis.

Interestingly, these same changes in relative cell growth were also observed when CDAP was reduced to 1 µM (see purple lines in Panels A and B). The reduced ability of CDAP (at 1 µM) to inhibit SMS significantly affected the composition of the polyamine pools. Indeed, the commitment to maintain spermine pools in the presence of DFMO (4.2 mM), DFMO + Spd, and DFMO + CDAP (1 µM) becomes obvious in Panel B. Both DFMO and DFMO + CDAP (1 µM) caused a significant decrease in relative cell growth (46.7% and 35%, respectively), which could be rescued to >90% relative cell growth by the availability of exogenous spermidine (1 µM). We noted that the presence of exogenous spermidine resulted in an approximate 2 nmol/mg protein increase in spermine levels (see red bars in Figure 5 Panel B). Interestingly, as seen in Panel A, when there is efficient inhibition of spermine biosynthesis, the intracellular spermidine pools were increased by 3 nmol/mg protein in the presence of exogenous spermidine (1 µM). These results suggest that polyamine import supplies at least 2–3 nmol/mg of spermidine to these cells, and when possible these spermidine resources are preferentially shunted to spermine pools. This preference for spermine is interesting because, as shown in the far right entries in Panels A and B (DFMO + CDAP + Spd), these cells can maintain high (>90%) relative growth in the presence of sufficient spermidine or spermine pools.

Given the impact of polyamine import on these results, we evaluated how the presence of a PTI (trimer44) affected inhibitor performance. The trimer44 PTI was shown to be a competitive inhibitor of polyamine transport via ^3^H-Spd uptake assays. Typically, as shown in Panel A, exogenous spermidine (Spd, 1 µM) was shown to rescue DFMO-treated L3.6pl cells from 46.7% relative growth with DFMO only (4.2 mM) to over 90% with DFMO + Spd. As shown in Figure 5 Panel C, the DFMO, DFMO + PTI, and DFMO + PTI + Spd treatments provided 46.7, 22.6, and 45.2% relative cell growth, respectively. In addition, the DFMO-only and DFMO + PTI + Spd entries had nearly identical polyamine pool distributions and % relative growth (46.7% and 45.2% in Figure 5, Panel C), illustrating the ability of the PTI to block both spermidine uptake and the rescue of DFMO-treated L3.6pl cells. The presence of the trimer44 PTI (4 µM) with DFMO + CDAP (100 µM) significantly lowered the cell growth to 6.8%. We also noted that the distribution of intracellular polyamine pools of DFMO + CDAP (100 µM) was similar to DFMO + CDAP (100 µM) + PTI + Spd, consistent with inhibited Spd import. In summary, only the therapies containing DFMO reduced cell growth at the concentrations used. This reduction in cell growth was augmented to some degree by the presence of CDAP and the PTI as single additives, and more so when they were used in combination with DFMO to provide extensive inhibition of polyamine metabolism.

In our titrations of CDAP with a fixed dose of DFMO (4.2 mM), we noticed a consistently lower relative cell growth (10%) in the presence of CDAP, even at concentrations where CDAP was not toxic. Indeed, this modest potentiation of DFMO was noted at as low as 100 nM CDAP, suggesting that even mild perturbation of spermine biosynthesis via SMS inhibition can augment the effectiveness of DFMO. Since DFMO + CDAP could be readily rescued by exogenous polyamines, the combination of these two agents may not be effective in vivo and may require the additional presence of the PTI for effective anti-proliferative activity.

## 4. Discussion

Of all three native polyamines, spermine has been shown to be the most effective immune suppressant, with inhibitory activity noted in T-cells, monocytes, and macrophages [14,15,16,17,18,19]. Compared to other human tissues, the human pancreas has the highest amount of spermidine. Armed with significant stores of spermidine, we hypothesized that PDAC tumors with upregulated SMS can convert spermidine to spermine (Figure 1) for immune suppression. Indeed, spermine is naturally present in amniotic fluid to suppress the maternal immune response and spermine has been shown to inhibit virtually all immune cells [14,15,16,17,18,19]. We speculated that PDAC uses this ‘fetal strategy’ to create a spermine-rich zone of immune privilege via spermine production and secretion. Rewardingly, a search of six existing pancreatic databases found that SMS mRNA is universally upregulated in PDAC, which is consistent with our hypothesis. This insight is potentially paradigm-shifting because it suggests that, unless spermine is downregulated in the PDAC tumor microenvironment, immunotherapies will continue to fail [20,21].

The results reported here suggest that even though PDAC cells can survive on either spermidine or spermine, they prefer spermine when given the choice (e.g., see DFMO results in Figure 5). This preference is consistent with the apparent high SMS expression in PDAC cells and may in part be critical for tumor survival by establishing immune privilege via the excretion of spermine or its metabolites.

Prior in vivo studies in healthy rats using MCHA showed that delivery was more effective via MCHA (0.1%, pH 6) in the drinking water than by a single intraperitoneal (i.p.) injection. A significant dose-dependent decrease in spermidine content was observed [10]. For example, using a 0.1% solution of MCHA for 10 days, Shirahata et al. observed a decrease in spermidine levels in prostate (28%), liver (21%), and kidney (33%) tissue with a concomitant increase in spermine levels. The total polyamine levels in these tissues did not fluctuate strikingly with the treatment, presumably due to polyamine import [10]. These results are very similar to what we observed with MCHA in culture with PDAC cells, where a shift in the intracellular polyamine distribution from spermidine to spermine occurs (Figure 4). The authors also reported the low toxicity of MCHA in rats with a lethal dose needed to kill 50% of the rats (LD_50_) of 250 mg/kg [10]. In summary, MCHA was effective in vivo in modulating spermidine pools in rat tissues.

Related studies in healthy rats with CDAP showed similar results to what we observed in cell culture, including a significant decrease in both spermine and putrescine pools, and a buildup of spermidine in the presence of CDAP [10]. For example, using a 0.1% CDAP solution in the drinking water for 10 days, the authors observed a 95% decrease in spermine content in the prostate and ~50% reduction in the liver and kidney tissues. Exposure to 0.1% CDAP in the drinking water showed a 30% decrease in spermine content in the liver of rats after 10 days and a 79% decrease in liver spermine levels and 211% increase in liver spermidine levels after 120 days of CDAP exposure. The authors also reported the low toxicity of CDAP in rats with a LD_50_ of 500 mg/kg [10]. In summary, CDAP was effective in vivo in modulating spermine pools in rat tissues and our in vitro results here are consistent with these prior findings in healthy rats.

While this is the first report of CDAP and MCHA assessment in pancreatic cancers, these compounds have been previously evaluated by He et al. in the treatment of P388 leukemia cells in mice [11]. Specifically, DFMO (1.5 g/kg) + CDAP (25 mg/kg) gave a 1.3-fold increase in survival [11]. The authors concluded that the anti-proliferative effect of DFMO was strengthened by CDAP due to a reduction in spermine content. When DFMO + CDAP was administered, a compensatory increase in spermidine was observed in P388 cells, but remained significantly lower than untreated P388 cells. Notably, the authors suggested that polyamine import may explain why, when CDAP was increased to 50 mg/kg, it failed to further decrease spermine levels [11]. In addition, when MCHA and CDAP were added individually with DFMO, only CDAP + DFMO significantly strengthened the antiproliferative effect of DFMO against leukemia cells [11]. This is interesting because we found in L3.6pl cells in vitro that both MCHA and CDAP can potentiate DFMO (i.e., further decrease cell growth) when used in combination with DFMO (Figure 4 and Figure 5).

In summary, many inhibitors of SRM and SMS have been developed, but few provide reduced cell viability [22,23,24,25]. One possible explanation is the observed increase in *S*-adenosylmethionine decarboxylase (SAMDC) activity when either spermidine or spermine intracellular stores are reduced [10]. Shirahata et al. demonstrated (albeit in the prostate) that either MCHA or CDAP, when dosed at 0.1% in the drinking water for 10 days, leads to the expected reduced levels of spermidine and spermine, respectively. MCHA (0.1%) and CDAP (0.1%) individually induced a 2.2-fold and 2.7-fold increase in SAMDC activity, respectively (see Figure 1) [10]. Indeed, depending upon the inhibitor used, the higher activity of SAMDC would lead to increased amounts of dc-SAM (i.e., *S*-adenosylmethioninamine) to facilitate the respective conversions of either putrescine to spermidine or spermidine to spermine as needed (Figure 1).

Therefore, in the presence of either MCHA or CDAP, the cells are likely primed for polyamine biosynthesis with high dc-SAM levels, but lack the specific polyamine substrates and non-inhibited enzymes needed to replenish their deficient pool(s). This explanation is consistent with the classic expectation that substrates buildup behind an enzymatic block. However, our results suggest that polyamine import plays a key role for how PDAC cells circumvent these inhibitors (by importing polyamine substrates like Spd) and suggest that the development of PTIs like trimer44 is warranted for future use in combination with DFMO or DFMO + CDAP. With the ability to decrease spermine and total polyamine pools in the presence of exogenous polyamines, the combination therapy of DFMO + CDAP + PTI provides a new way to target PDAC cells via their altered polyamine metabolism.

## 5. Conclusions

Understanding how PDAC cells respond to inhibitors of polyamine metabolism and import could lead to new approaches to treat PDAC. For example, combination therapies like DFMO + CDAP + PTI, which reduce intratumoral spermine pools, even in the presence of exogenous polyamines, may provide a new approach to PDAC tumors by targeting their high reliance upon spermine. Reduced spermine production by the tumor would result in less spermine available for secretion to establish immune privilege. Indeed, others have shown that DFMO + PTI therapies provide an enhanced immune response in other cancers [26,27,28]. While the precise genes and proteins involved in polyamine transport are only now coming to light [29], this area is ripe for future drug discovery. The findings that DFMO and CDAP are both orally bioavailable, have low toxicity, and CDAP levels even as low as 100 nM in culture provide beneficial potentiation of DFMO, provide additional rationale for continued studies of these interventions in PDAC.

## Figures and Tables

**Table 1:** Polyamine levels as a function of DFMO concentration in L3.6 pl cells incubated at 37 °C for 72 h ^a^.

Experiment	DFMO (mM)	Spermidine nmoles/mg Protein	Spermine nmoles/mg Protein	Total Polyamines nmoles/mg Protein	Spd/Spm Ratio
Control	0	13.0 ± 1.6	10.1 ± 0.9	23.1	1.29
DFMO	0.1	12.0 ± 2.8	13.4 ± 4.3	25.3	0.90
DFMO	0.5	0.7 ± 0.1	15.3 ± 2.3	16.1	0.05
DFMO	1	0.2 ± 0.0	14.9 ± 0.8	15.2	0.01
DFMO	2	0.6 ± 0.7	9.2 ± 3.9	9.8	0.06
DFMO	3	0.1 ± 0.0	6.1 ± 0.2	6.2	0.01
DFMO	4.2	0.2 ± 0.0	7.7 ± 1.0	7.9	0.02

^a^ error is listed as standard deviation around the mean. Note the putrescine level for the control was 0.8 ± 0.6 nmoles/mg protein and was undetectable after DFMO addition.

**Table 2:** Polyamine levels as a function of trimer44 concentration in L3.6pl cells incubated at 37 °C for 72 h ^a^.

Experiment	trimer44 (µM)	Spermidine nmoles/mg Protein	Spermine nmoles/mg Protein	Total Polyamines nmoles/mg Protein	Spd/Spm Ratio
Control	0	14.0 ± 3.6	8.5 ± 1.8	22.5	1.65
trimer 44	1	17.0 ± 6.3	12.9 ± 3.4	29.9	1.31
trimer 44	2	12.2 ± 2.8	8.9 ± 1.2	21.1	1.36
trimer 44	4	12.0 ± 5.3	10.4 ± 2.1	22.4	1.15
trimer 44	5	11.4 ± 2.5	13.5 ± 0.8	24.9	0.84
trimer 44	10	5.5 ± 1.5	11.2 ± 2.1	16.7	0.49

^a^ error is listed as standard deviation around the mean.
